# Determination of Nitric Oxide-Derived Nitrite and Nitrate in Biological Samples by HPLC Coupled to Nitrite Oxidation

**DOI:** 10.1007/s10337-013-2529-0

**Published:** 2013-08-04

**Authors:** Anguo Wu, Tingting Duan, Dan Tang, Youhua Xu, Liang Feng, Zhaoguang Zheng, Jiaxiao Zhu, Rushang Wang, Quan Zhu

**Affiliations:** 1 State Key Laboratory of Quality Research in Chinese Medicine, Faculty of Chinese Medicine, Macau University of Science and Technology, Macau, People’s Republic of China; 2 Institute of Consun Co. for Chinese Medicine in Kidney Diseases, Guangdong Consun Pharmaceutical Group, 71 Dongpeng Road, Guangzhou, 510530 People’s Republic of China; 3 Key Laboratory of Delivery Systems of Chinese Meteria Medica, Jiangsu Provincial Academy of Chinese Medicine, Nanjing, People’s Republic of China

**Keywords:** HPLC, Nitrite, Nitrate, Nitric oxide, Acid potassium permanganate

## Abstract

Nitrite and nitrate are main stable products of nitric oxide, a pivotal cellular signaling molecule, in biological fluids. Therefore, accurate measurement of the two ions is profoundly important. Nitrite is difficult to be determined for a larger number of interferences and unstable in the presence of oxygen. In this paper, a simple, cost-effective and accurate HPLC method for the determination of nitrite and nitrate was developed. On the basis of the reaction that nitrite is oxidized rapidly to nitrate with the addition of acidic potassium permanganate, the determination of nitrite and nitrate was achieved by the following strategy: each sample was injected twice for HPLC analysis, i.e. the first injection was to measure nitrate, and the second injection was to measure total nitrate including initial nitrate and the nitrate from the conversion of nitrite with the addition of acid potassium permanganate in the sample. The amount of nitrite can be calculated as difference between injections 2 and 1. The HPLC separation was performed on a reversed phase C_18_ column for 15 min. The mobile phase consisted of methanol–water (2:98 by volume); the water in the mobile phase contained 0.60 mM phosphate salt (potassium dihydrogen and disodium hydrogen phosphate) and 2.5 mM tetrabutylammonium perchlorate (TBAP). The UV wavelength was set at 210 nm. Additionally, we systemically investigated the effects of the concentration of phosphate salt and TBAP in the mobile phase, the pH of the mobile phase, and the amount of acidic potassium permanganate added to the sample on the separation efficacy. The results showed that the limits of detection (LOD) and the limit of quantitation (LOQ) were 0.075 and 0.25 μM for nitrate (containing the oxidized nitrite), respectively. The linear range was 1–800 μM. This developed approach was successfully applied to assay nitrite/nitrate levels in cell culture medium, cell lysate, rat plasma and urine.

## Introduction

Nitric oxide (NO) is both a gaseous free radical and a bioactive molecule that plays important roles in the regulation of vascular tone, immune system function, neurotransmission, and so on [1]. NO is biosynthesized from the amino acid l-arginine by nitric oxide synthase (NOS) in vascular endothelial cells [2]. Direct measurement of NO in plasma, serum, and urine is difficult due to its short half-life of NO in vivo (<0.1 s) [3]. Evaluating the body formation of NO is to measure its more stable oxidation products nitrate and nitrite in biological fluids [4].

Extensive interests have been shown to determine nitrite and nitrate in biological fluids. The methods include Griess reaction [5], chemiluminescence [6], fluoelectrochemical probes [7], spectrophotometry [8], electron paramagnetic resonance [9], electrophoresis [10] high-performance liquid chromatography [11], gas chromatography [12], and ion chromatography [13]. Among these reported methods, the HPLC methodology with the advantages of high sensitivity was widely applied. However, most HPLC methods are frequently applied to measure nitrite in biological samples such as plasma or urine due to considerable interference from the big negative peak of the larger amounts of chloride preceding nitrite, which often masks the nitrite peak. Additionally, some of these methods were based on the conversion of nitrate to nitrite and the derivatization of nitrite with other reagents, so the sample pre-treatment was somewhat complicated. Furthermore, the reduction product, nitrite or NO is unstable in the presence of oxygen.

In the present study, we developed a new simple and effective HPLC method for determining nitrite and nitrate in most of biological samples. On the basis of the reaction that nitrite is oxidized rapidly to nitrate with the addition of acidic potassium permanganate, the determination of nitrite and nitrate was achieved by a following strategy: each sample was injected twice for HPLC analysis, i.e. the first injection was to measure nitrate, and the second to determine total nitrate including initial nitrate and the nitrate from the conversion of nitrite with the addition of acid potassium permanganate in the sample. The amount of nitrite can be calculated as difference between injections 2 and 1. Furthermore, this method was used to measure nitrite and nitrate levels in cell culture medium, cell lysate, rat plasma, and urine.

## Experimental

### Chemicals

Deionized water was prepared using a Milli-Q^®^ (Millipore, Molsheim, France) system. Potassium permanganate, potassium dihydrogen phosphate, disodium hydrogen phosphate, sulfuric acid, potassium nitrate, and sodium nitrite of analytical grade were purchased from Tianjin Damao Chemical Reagent Factory (Tianjin, China). Tetrabutylammonium perchlorate (TBAP) was obtained from Aladdin Chemistry Co. (Shanghai, China). Methanol was from Merck (Hohenbrunn, Germany), Dulbecco’s Modified Eagle’s Medium (DMEM) was obtained from Hyclone (Logan, Utah, USA). All other chemicals were at least analytical grade.

### Solutions

3.45 mg of potassium nitrate and 5.05 mg of sodium nitrite were dissolved together using 50 mL deionized water and the final concentration was 1 mM. For the acidic potassium permanganate solution, equal volumes of dilute (about 2 mol L^−1^) sulfuric acid and dilute (0.02 mol L^−1^ or less) potassium permanganate solution were mixed, and the final concentration was 0.01 mol L^−1^. Because the acidic potassium permanganate solution is unstable, it should be prepared just before use.

### Chromatography

The chromatograph comprised a Shimadzu 10AVP sample controller, two LC-ADVP solvent-delivery units, SIL10ADVP autosampler, SPD 10 AVP photo diode array detector (PDA) set at 210 nm and a CLASS-VR^®^ workstation (Shimadzu, Tokyo, Japan), and a reversed phase C_18_ column (250 × 4.6 mm, 5 μm) guarded by a 10-μm reversed-phase C_18_ column, which were all purchased from Agilent Technologies, Inc. (Palo Alto, CA, USA). The mobile phase was pumped at a rate of 1 mL min^−1^ and consisted of methanol-water (2:98 by volume); the water in the mobile phase contained 0.60 mM of phosphate salt (potassium dihydrogen and disodium hydrogen phosphate) and 2.5 mM TBAP.

### Sample Preparation

#### Cell Culture Medium and Cell Lysate

Endothelial cells were cultured at 37 °C in DMEM in a humidified CO_2_/air atmosphere. After 48 h of culture period, the conditioned medium and the cell lysate were collected and and 2 mL ethanol added. These solutions were centrifuged at 5,000 rpm for 10 min after standing at −20 °C for 30 min. The clean supernatants were concentrated under vacuum; then the dried samples were redissolved in 250 μL deionized water and immediately analyzed by HPLC.

#### Rat Plasma and Urine

1 mL samples of blood and urine were collected from the fasting rat. The samples were precipitated by adding 0.25 mL ethanol and centrifuged at 5,000 rpm for 10 min after standing at −20 °C for 30 min. The supernatants were concentrated under vacuum freeze-drying, and then the dried samples were redissolved in 250 μL deionized water.

## Results

### Optimization of Chromatography Conditions

In this experiment, we selected culture medium DMEM as the analysis subject for optimizing the chromatography conditions.

#### Effect of Phosphate Salt

**Fig. 1:**
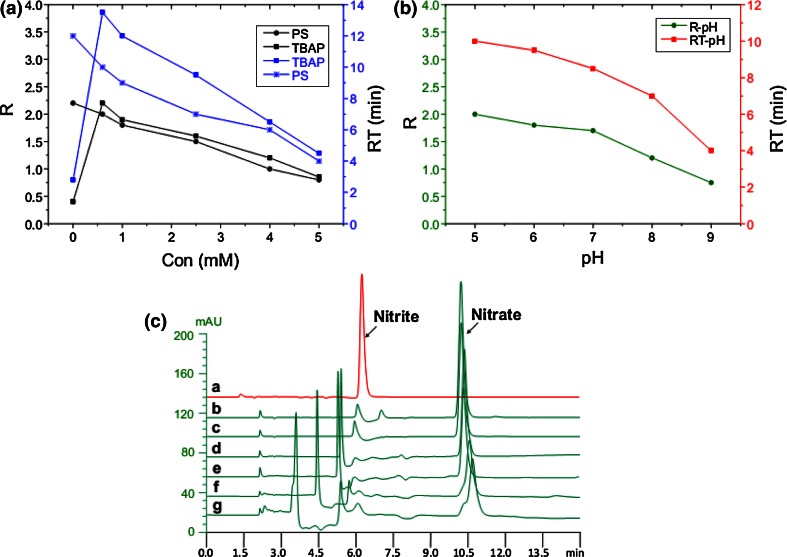
The optimization of mobile phase condition and the concentration of oxidation reagent. **a** The effects of phosphate salt (PS) and TBAP on the resolution (R) and the retention time (RT) of nitrate. The *black Y*-axis and curve represent the effect of PS and TBAP on R, and the *blue Y*-axis and curve represent the effect of PS and TBAP on RT. **b** The resolution and the retention time of nitrate declined with pH. **c** A greater amount of acidic potassium permanganate was added to the solutions, and the worse of the peak shape of nitrate was generated (*a*–*g* represent different volumes of acidic potassium permanganate were added into 250 μL of the solution with nitrite). *a*: 1 μL; *b*: 2 μL; *c*: 4 μL; *d*: 10 μL; *e*: 20 μL;* f*: 40 μL; *g*: 80 μL

We have investigated the effect of the concentrations (0–5 mM) of phosphate salt in water of the mobile phase on the nitrate separation in DMEM. The concentration of TBAP was fixed at 2.5 mM. Figure 1a shows that the higher the concentration of phosphate salt in the range 0–5 mM, the shorter the retention time but the worse of resolution of nitrate. Meanwhile, when the retention time was about 4 min, the nitrate peak was tended to be interfered by the other peaks. Furthermore, higher concentration would result in blocking the instrument and shortening the column life. Therefore, we selected 0.6 mM of phosphate salt in this experiment after considering all the factors.

#### Effect of TBAP

TBAP as the ion-pair reagent has the function of extending the retention time of ions in HPLC and improve the peak shape. In this experiment, the retention time of nitrate extended and the peak separated from other peaks without any interference. The effect of TBAP with the concentrations (0–5 mM) on the separation was also studied. We fixed the concentration of phosphate at 0.6 mM according to the above data. From Fig. 1a, we concluded the effect was similar to that of phosphate salt when the concentration range was 0.5–5 mM. When the concentration of TBAP increased from 0 to 0.5 mM, the retention time became longer. When the concentration of TBAP was 5 mM or more, the dissolution of it in water needed much more time before the analysis, and the column need a longer time to wash after the analysis. Furthermore, the higher concentration of TBAP and the shorter retention time of nitrate would happen. Meanwhile, when the concentration was lower (e.g. 0.5 mM), it would suffer from the disadvantage of long balance time (>2 h) before beginning the analysis, and accordingly, completing one analysis needed a longer time. Finally, we set the concentration of TBAP at 2.5 mM after comprehensively considering the factors including both the resolution and analytic time.

#### Effect of pH

The mobile phase pH plays an important role in the analysis, and it can affect the retention time, column efficiency, and life. In this paper, we have studied different pH values (5, 6, 7, 8, and 9) adjusted with NaOH solution or phosphoric acid. According to the above results, the concentrations of phosphate salt and TBAP were set at 0.6 and 2.5 mM, respectively. Figure 1b shows the retention time and resolution declined with pH. Taking various factors into consideration, we selected pH as 5–7 for this analysis. Interestingly, we found that the pH of the deionized water containing 0.6 mM phosphate and 2.5 mM TBAP was just 6.0–6.5 without adjustment.

#### Effect of Oxidation Reagent

The acidic potassium permanganate solution was prepared according to paragraph 2.2.1. Different volumes (1–80 μL) of the oxidation reagent (0.02 mol L^−1^) were added to 250 μL of nitrite standard solution (0.5 mM). We found that only 2 μL of the oxidation reagent could completely oxidize nitrite in standard solution or samples to nitrate in 1 min (showed in Table 1). The permanganate/thiosulphate titrimetric method is used to determine nitrite and find that the blue color disappears for at least 3 s (EuSalt/AS 001-2005), which suggests that this reaction is fast on the condition that the presence of enough acidic potassium permanganate. The oxidation rate and the number of theoretical plates (NTP) were investigated (peak shapes showed in Fig. 1c), and the more of the oxidation reagent added, the smaller the NTP and the worse the peak shape. There was no influence on the peak shape as the addition was the range of 2–10 μL. When the addition was more than 40 μL, the NTP declined 40 % at least, which affected seriously the peak shape of nitrate. Furthermore, the greater the concentration of oxidation reagents added, the more numerous peaks interfered with nitrate.

**Table 1:** Reaction time of acidic potassium permanganate with nitrite

Reaction time	20 s	40 s	1 min	2 min	5 min
Nitrite (0.5 mM) in standard solution (%)	97	99	100	100	100
Nitrite (5.76 μM) in plasma (%)	99	100	100	100	100

### Method Validation

#### Linearity

**Fig. 2:**
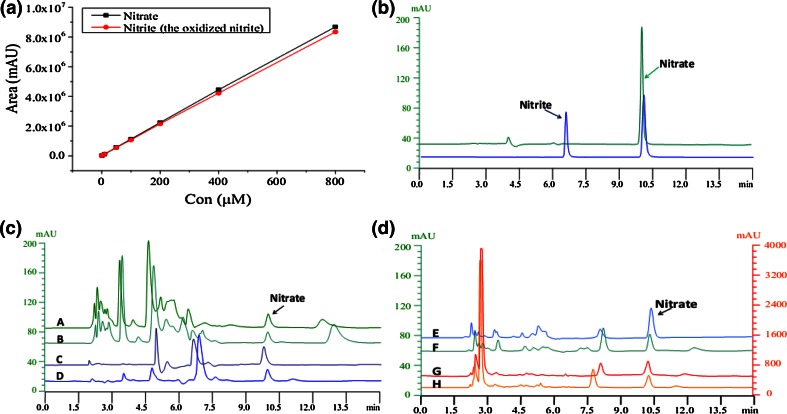
Calibration graphs and the typical chromatograms of the standard solution and the samples. **a** The calibration graphs. **b** The mixture of nitrite and nitrate in aqueous solution (the *blue* chromatogram) was added to acidic potassium permanganate and then nitrite was oxidized into nitrate (the *green* chromatogram). **c** The chromatograms of DMEM and cell lysate with their corresponding oxidized samples. *A*: DMEM which was added with acidic potassium permanganate (the second injection); *B*: DMEM which was not added with acidic potassium permanganate (the first injection); *C*: cell lysate which was added with acidic potassium permanganate (the second injection); *D*: cell lysate which was not added with acidic potassium permanganate (the first injection). **d** The chromatograms of plasma and urine with their corresponding oxidized samples. The *Y*-axis on the left corresponds to the plasma, and the *Y*-axis on the right represents the urine. *E*: Plasma which was added with acidic potassium permanganate (the second injection); *F*: Plasma which was not added with acidic potassium permanganate (the first injection); *G*: Urine which was added with acidic potassium permanganate (the second injection); *H*: Urine which was not added with acidic potassium permanganate (the first injection)

The calibration graphs were constructed by plotting the peak areas against the concentrations of the mixed standard of potassium nitrate and sodium nitrite (the oxidized nitrite) (Fig. 2a). The concentrations were set at 1, 10, 50, 100, 200, 400, and 800 μM, respectively. Plotting the graph with the peak areas of nitrate and nitrite (the oxidized nitrite) as the dependent variable (*Y*-axis) and concentration as the independent variable (*X*-axis) results in the equations as follows: *Y* = (10862.33 ± 1.52)*X* + (24655.67 ± 1161.92), *R* ^2^ = 0.9998 for nitrate, and *Y* = (10416 ± 4)*X* + (25877.33 ± 787.77), *R* ^2^ = 0.9999 for nitrite (the oxidized nitrite), which was calculated by the method of calculating difference in value from the two injections. The chromatograms of the standard solution and the relative oxidized solution were showed in Fig. 2b. Linearity was addressed by preparing seven standard solutions of nitrate and nitrite (the oxidized nitrite) ranging between 1 and 800 μM.

#### Repeatability, Precision, and Recoveries

The repeatability of this method was evaluated by injecting six independently prepared mixed standard solutions that contain nitrite and nitrate as well as the solution added the acidic potassium permanganate solution. The results were presented in Table 2. The intra-day and inter-day variabilities of each peak of the solutions were examined for investigating the precision. Meanwhile, the intra-assay was performed with the interval 1 h for 6 h, and the inter-assay was performed over 2 days. The results listed in Table 2 showed that the RSD values of both retention time and peak area were lower than 0.6 %. The recovery test was performed to examine the accuracy of the oxidization method, samples spiked with appropriate amounts of nitrite and nitrate, and the spiked amount was adjusted so as to provide a concentration similar to that present in the sample. For the recovery % of nitrite, nitrite in the standard solution (1) and in the biological samples (2) was determined by HPLC coupled with the nitrite oxidation according to this method; then nitrite solution was added to the biological samples and the concentration of nitrite determined (3). The recovery % of nitrite was achieved as follows: recovery (%) = 100 × (amount found_3_ − original amount_2_)/amount spiked_1_. For the recovery % of nitrate, the procedure was the same as nitrite. Nitrate was determined by HPLC without any sample pre-treatments according to this method.

**Table 2:** Repeatability and precision of this method

Compounds	Repeatability		Precision (intra-day)		Precision (inter-day)	
	RSD of retention time (%)	RSD of peak area (%)	RSD of retention time (%)	RSD of peak area (%)	RSD of retention time (%)	RSD of peak area (%)
Nitrite	0.04	0.29	0.05	0.31	14	0.52
Nitrate	0.08	0.23	0.09	0.28	0.12	0.38
Nitrate (the oxidized nitrite)	0.02	0.25	0.08	0.29	0.13	0.46

#### Limit of Detection (LOD) and Limit of Quantification (LOQ)

The LOD of nitrate and nitrite (the oxidized nitrite) was 0.075 μM, while 0.1 μM for the unoxidized nitrite. Both of their LOQ was 0.25 μM, but 0.33 μM for the unoxidized nitrite, which further demonstrated that the developed method based on the conversion of nitrite to nitrate by oxidation for quantitative analysis exhibited better sensitivity.

### Analysis of Sample

For analysis of nitrite and nitrate in the DMEM, plasma and urine, the peaks of nitrate from the first injection correspond to nitrate concentration, while the peaks of nitrate from second injection correspond to the total nitrate including the nitrate from the first injection and the oxidized nitrite. Before the second injection, 250 μL of the sample was added with 5 μL acidic potassium permanganate solution and kept for 1–5 min. The analysis reached completion within 15 min. As shown in the Fig. 2, the chromatograms of B, D, F, and H were from the first injections, while those for A, C, E, and G were from the second injections. All the chromatograms showed that there was no interference around nitrate (Fig. 2). The concentrations of nitrite and nitrate in cell culture medium, cell lysate, plasma, and urine were presented in Table 3. The RSD values of both accuracy and reproducibility of all were <5 %.

**Table 3:** The concentrations and recoveries of nitrite and nitrate in DMEM, cell lysate, plasma and urine by this HPLC method

Sample	Concentration (μM)		Recovery (%) (*n* = 6)	
	Nitrate	Nitrite	Nitrate	Nitrite
DMEM	6.46	1.51	100.43 ± 1.32	99.82 ± 3.56
Cell lysate	1.72	0.47	99.18 ± 2.31	100.54 ± 1.67
Plasma	6.08	5.76	99.43 ± 1.45	101.62 ± 0.23
Urine	124.01	5.60	101.23 ± 0.94	99.65 ± 2.53

## Discussion

Nitrite and nitrate in body fluids are from in vivo and in vitro. Nitrite and nitrate are mainly accumulated as a result of NO production through NOS enzymes in vivo. For in vitro, cell culture studies deal with NO production and consumption, which involve culturing different types of cells and evaluating various types of drugs, enzymes, regulators, and inhibitors. The cell culture medium contains many complicated components; therefore, the analysis of nitrite and nitrate seems to be difficult. Although nitrite and nitrate are mainly derived from NO in vivo, the nitrogen oxide gas, conventional diet, drugs, and many supplements are also the source in vitro [14]. All nitrite and nitrate are then excreted in urine through the kidney. There are more amounts in urine than in other body fluids, and nitrate is the predominant one [15] (we can also get this conclusion from the results). Although nitrite is a major end product of nitrogen monoxide metabolism, it cannot accumulate in vivo and is rapidly oxidized by oxyhemoglobin or oxymyoglobin to form methemoglobin or metmyglobin and nitrate which leads to less amount compared with nitrate. Nitrite in the collected biological samples can also be slowly oxidized to nitrate in the presence of oxygen. For these reasons, we should determine the total amounts of nitrite and nitrate in biological samples to evaluate the NO levels. In this experiment, the total contents of nitrite and nitrate represent NO levels.

Some of the reported HPLC methods were based on the conversion of nitrate to nitrite or the derivatization of nitrite and nitrate with other reagents [6, 7, 11]. However, their sample preparations were complicated. Furthermore, nitrite is less stable than nitrate and could be oxidized to nitrate slowly. In this paper, as shown in the Fig. 2, the peak of nitrite was interfered seriously by other compounds; for this reason, based on the reaction of nitrite with a small amount of acidic potassium permanganate to form the more stable nitrate completely in 1–5 min, we developed a new method for converting nitrite to nitrate and applying HPLC to measure nitrite and nitrate. This assay was successfully applied to detect nitrite and nitrate in DMEM with many complicated components. Mobile phase pH, the concentration of phosphate salt, and TBAP in the water greatly influenced the separation of nitrate. The optimal conditions were pH with 6.0–6.5 without any adjustment, 0.60 mM phosphate salt and 2.5 mM TBAP. At the same time, this method can be used to determine nitrate in plasma and urine. The measurement of nitrite and nitrate could reflect the levels of NO in biological fluids. The accuracy and reproducibility showed that this method was good for determining nitrite and nitrate in biological samples.

## Conclusion

In conclusion, although there were many HPLC methods used for the determination of nitrite and nitrate, for the first time, we evaluated NO levels by measuring the amount of nitrite and nitrate based on the conversion of nitrite to nitrate in various biological fluids such as cell culture medium, cell lysate, plasma and urine in rat. Easy sample preparation and absence of expensive agents and time-consuming procedures make this approach a useful tool for investigating NO synthesis in vitro and in vivo under physiological and pathophysiological conditions. Our HPLC method for nitrite and nitrate analysis may greatly facilitate research in the ever-expanding field.
